# No simple way to averaging out: Pooled mesenchymal stromal cells do not reflect average donor characteristics

**DOI:** 10.1016/j.reth.2025.09.012

**Published:** 2025-10-03

**Authors:** Dea Kukaj, Sabine Niebert, Christoph Biehl, Ursula Reichart, Christiane Schueler, Janina Burk

**Affiliations:** a Physiology & Pathophysiology, Department of Biological Sciences and Pathobiology, University of Veterinary Medicine Vienna, Vienna, Austria; b Equine Clinic, Faculty of Veterinary Medicine, Justus-Liebig-University, Giessen, Germany; c Department of Trauma, Hand and Reconstructive Surgery, University Hospital of Giessen, Justus-Liebig-University, Giessen, Germany; d VetCore Facility for Research/Imaging Unit, University of Veterinary Medicine Vienna, Vienna, Austria

**Keywords:** mesenchymal stromal cells, human MSCs, adipose-derived, donor variability, heterogeneity, cellular fitness, cell pooling, functional characterization, cell tracking

## Abstract

**Background:**

Mesenchymal stromal cells (MSCs) are promising candidates for numerous regenerative therapies. Still, clinical translation is complicated by the heterogeneity of MSCs and related shortcomings in preclinical research. Pooling MSCs from multiple donors is increasingly being advocated as an effective way to mitigate donor variability. However, it remains unclear whether the range of individual cell characteristics is equally reflected in pooled cultures, or if pooling rather leads to a homogenized cell population dominated by the fittest donor, which would lead to skewed results. This study investigates whether MSC pools are functionally representative for their respective donor MSCs and whether dominant donors emerge over time.

**Methods:**

MSCs from nine human donors were categorized into low-, middle-, and high-fitness groups. Individual MSCs were then pooled according to their fitness groups, complemented by a mixed-fitness pool. Functional assays for proliferation, metabolic activity, differentiation, migration and senescence were performed to evaluate the pools versus the individual MSCs. Donor representation within pools was tracked using fluorescence microscopy and qPCR.

**Results:**

The high-fitness pool, as well as its individual donor MSCs, displayed the most rapid proliferation and highest metabolic activity. However, while for proliferation, the pool data aligned well with the individual donor data, all other assays revealed discrepancies between the pooled cultures and individual donor cells. Interestingly, particularly the mixed fitness pool showed inferior metabolic activity and differentiation potential in comparison with the respective individual donor MSCs. Cell tracking showed that over one passage, even pools composed of donors with similar cell fitness became dominated by the donor with the highest cellular fitness.

**Conclusions:**

The discrepancy between pooled and individual donor data emphasizes the importance of biological replicates to capture donor variation and ensure that MSC research reflects natural diversity.

## 1 Introduction

Mesenchymal stromal cells (MSCs) are of mesenchymal origin and characterized by their ability to adhere to plastic surfaces, differentiate into osteogenic, chondrogenic, and adipogenic lineages, and express the surface antigens CD73, CD90, and CD105 [1]. MSCs can be isolated from various tissues, including bone marrow [1], adipose tissue [2], umbilical cord [3], Wharton's jelly [4], endometrium [5], dental pulp [6], and menstrual blood [7].

Due to their multifaceted modes of action and relative ease of collection and expansion, MSCs are considered highly promising for clinical applications in diseases of the immune system, internal organs, and musculoskeletal system alike [[8], [9], [10], [11], [12]]. However, despite being in the focus of many research projects, there remains a significant discrepancy between the numerous preclinical studies and early clinical trials versus the very few phases III and IV trials and approved cell products [8,[13], [14], [15], [16]]. As of July 2025, current data provided by ClinicalTrials.gov show that out of a large number of ongoing MSC-based clinical trials, merely three studies have progressed to phase IV. This disparity is partially attributed to the limitations of current preclinical models, which often fail to adequately reflect the complexity of real-world conditions, making the efficacy of MSCs in vivo difficult to predict [17,18].

While advancements in cell culture systems have improved their physiological relevance, one pivotal factor remains difficult to handle: the individuality of MSCs from different donors. Donor-dependent variability is well-documented [[19], [20], [21]] and may lead to variable potency and therapeutic efficacy. However, there is no common ground on how this should be reflected in basic research and preclinical models or handled in clinical scenarios.

Incorporating the primary cells from different donors as biological replicates in the study design, i.e. performing experiment repetitions with cells from different donors separately, is the straightforward way to reflect the biological variation among cells from different donors [22]. Yet, while entailing insight into the full range of possible cell characteristics, this approach comes with widespread data, which is unpopular as it prevents potential differences between treatment groups from reaching “statistical significance”. Similarly, batch-to-batch consistency between MSCs derived from different donors for clinical application is poor, hampering the standardization of treatment protocols and consistent treatment success. Therefore, approaches to overcome the variability issue related to biological replicates and single-donor MSC batches appear favorable.

Pooling of cells from different donors and then performing experiment repetitions with this cell pool, i.e. as (independent) technical replicates, is a typically used strategy for small animal primary cells, where cells from several (homogeneous) donors are combined to obtain suffient cells. Pooling is also used in human MSC research, yet with inconsistent reporting practices, and has specifically been proposed by some research groups and companies as a way to reduce data variability and standardize MSC-based therapies [23,24]. Furthermore, some studies have fostered hope that potency could be increased by pooling, having observed a higher immunosuppressive potential of pooled vs. individual donor MSCs [23,25]. Although the latter was not confirmed in other studies [26,27], pooling is generally a conceivable and attractive approach - which, however, still suffers from several shortcomings.

First, transparency regarding the use of this approach in human MSC research is poor. Among current PubMed-listed articles on in vitro studies with human primary MSCs, only some clearly describe whether the data shown were obtained with biological replicates or with technical replicates from pooled MSCs. When the use of pooled MSCs was disclosed, the number of donors or the pooling approach were not always specified. This widespread lack of transparency complicates the interpretation of experimental findings and impedes efforts to replicate or compare results across studies.

Second, batch-to-batch variability is not necessarily reduced by pooling. Indeed, MSC pooling reduces experimental variability and allows for more consistent results among the technical replicates obtained from one particular cell pool as compared to biological replicates, as demonstrated previously [28]. Yet importantly, it is already evident from previous studies that different MSC pools do not yield the same results [[29], [30], [31]]. Therefore, so far, the rationale for testing pooled MSCs for clinical therapies is mainly that higher cell numbers are available without extensive passaging, but not that the therapeutic product is more consistent between batches.

Third, representing the main rationale for the current study, in basic MSC research, the pooling approach has to assume that the pooled cell population represents the properties of all donors equally. Otherwise, experiments would not be representative for the heterogeneous MSC properties in different donors. Unfortunately, this is a notion that lacks evidence. In contrast, it has already been shown that MSC pools, compiled at passage 1, undergo changes in their composition until passages 3 and 5 [31]. Thus, it is questionable in how far all donors are still functionally represented by pooled cell populations, and whether the pooling strategy is suitable to replace biological replicates.

In this study, we sought to shed more light on the characteristics and composition of pooled MSC samples, with different MSC pools being compiled based on the individual donors' MSC fitness levels. We hypothesized that cell pools composed from donors with heterogeneous cellular fitness would rapidly be dominated by the fittest individual cells and therefore display a higher level of functionality than the individual donors’ average, leading to a distorted impression of average MSC potency.

## 2 Materials and methods

### 2.1 Study design

**Fig. 1:**
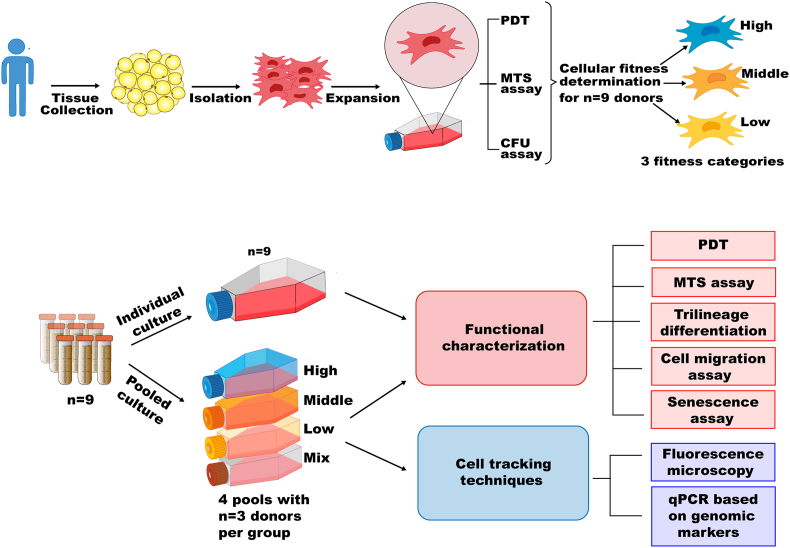
Study design.

Primary MSCs from different human donors were first subjected to assays characterizing their cellular fitness and thereby categorized into low, middle and high fitness groups. MSCs from n = 9 donors were then selected for the subsequent part of the study, at which low, middle, high and mixed fitness MSC pools, each consisting of n = 3 donors' MSCs, were composed. These different MSC pools were on the one hand subjected to functional assays in parallel to the respective individual MSCs and on the other hand monitored with respect to their composition and thus, the possible dominance of specific donors over time (Fig. 1).

### 2.2 MSC isolation and culture

Adipose-derived MSCs were either isolated in-house or obtained from a commercial supplier (PromoCell, Heidelberg, Germany; lot numbers: 471Z028, 428Z005.3, 476Z008, 409Z019, 479Z011.3, 485Z035). For in-house cell isolation, waste material tissues from orthopedic surgeries were obtained from the Clinic of Orthopedics, University Clinical Center of Giessen, Germany, with the donors' written informed consent (ethics commission approval AZ 209/21). The cells were isolated and expanded using standard protocols described previously and cryopreserved [32]. Commercially sourced cells were thawed and cultured according to the manufacturer's recommendations. All cells were then aliquoted and cryopreserved at passage 1 or 2.

### 2.3 Characterization of cellular fitness

Aliquots of the cells from all donors were thawed and subjected to a set of assays to determine the cellular fitness for each donor, which served as a basis for selecting three donors for each fitness group.

#### 2.3.1 Population doubling assay

Days to reach confluence were recorded to calculate the population doubling time (PDT) as a measure of proliferative potential. Cells were seeded at a density of 3000 cells/cm^2^ and checked daily. After reaching >80 % confluency, as documented by phase contrast microscopy and measured with ImageJ software, cells were detached and reseeded at the same density. The procedure was continued until the 10th passage. The PDT was calculated at each passage using the formula:

PDT=daysinculture/[(LN(no.ofcellsharvested/no.ofcellsseeded))/LN(2)]

#### 2.3.2 Metabolic activity assay

The metabolic activity of the cell cultures was assessed using an MTS assay (CellTiter 96® AQueous One Solution Cell Proliferation Assay, Promega GmbH, Walldorf, Germany) according to the manufacturer's instructions, with absorption measurements on day 4 after seeding.

#### 2.3.3 Colony forming unit assay

To measure the presence of cells capable of clonal expansion, cells were seeded in 6-well plates at a density of 20 cells/cm^2^, left in culture for two weeks, stained with Crystal Violet (Merck KGaA, Darmstadt, Germany). The number of colonies was counted using ImageJ software and plating efficiency (PE) was calculated using the formula:

PE(%)=no.ofcolonies/no.ofcellsseeded∗100

#### 2.3.4 Fitness scoring and donor demographics

Integrating the results from these assays, a fitness score was calculated for each donor's MSCs. The assay results and the corresponding scorings are detailed in the Supplementary Material (Table S1 and S2). The total fitness score was then used to assign the donors to their respective fitness group, based on which the low, middle, high and mixed fitness MSC pools were composed in the subsequent part of the study. The demographics and cellular fitness groups for the donors included in the pools are presented in Table 1.

**Table 1:** Donor demographics and corresponding cellular fitness groups.

Donor	Age	Sex	Total fitness score	Cellular fitness group
1	89	F	9	Low∗
2	78	F	13	Low
3	57	F	12	Low
4	76	F	16	Middle
5	46	F	15	Middle∗
6	30	F	14	Middle
7	38	F	17	High
8	29	M	18	High∗
9	40	F	26	High

M: male; F: female. Age is shown in full years. Donors were categorized into low, middle, and high cellular fitness groups. One donor from each fitness group was additionally included in the mixed cellular fitness pool (marked with an asterisk).

### 2.4 Functional characterization of MSCs in individual and pooled cultures

Fresh aliquots of cells were thawed and cultured using Mesenchymal Stem Cell Growth Medium 2 (PromoCell, Heidelberg, Germany) according to the manufacturer's instructions. When >80 % confluency was reached, they were harvested with Accutase™ solution (Merck KGaA, Darmstadt, Germany) and counted manually with a Neubauer hemocytometer, using Trypan blue staining to exclude dead cells.

The MSCs (passage 2 or 3) were then reseeded in individual and pooled cultures. For the pooled cultures, cells from the three donors included in the respective pool were combined equally (1:1:1). The following experiments were performed simultaneously in the individual and pooled cultures.

#### 2.4.1 Population doubling assay

Cells were seeded at a density of 3000 cells/cm^2^, detached on day 5, counted, and PDT was calculated using the formula stated above.

#### 2.4.2 Metabolic activity assay

The MTS assay was also performed again in this part of the study for both individual and pooled cultures, using the same reagent and procedure as described above and with measurements on days 1, 4 and 7.

#### 2.4.3 Trilineage differentiation

The cells were tested for their trilineage differentiation capacity. For osteogenic and adipogenic differentiation, the cells were seeded at 2000 and 3000 cells per well in 24-well plates, respectively. On day 3, the standard culture medium was replaced with the respective differentiation medium provided by the StemPro™ Differentiation Kits (Thermo Fisher Scientific GmbH, Dreieich, Germany). Chondrogenic differentiation was performed in 3D pellet cultures, which were prepared by centrifuging 500,000 cells suspended in StemPro™ chondrogenic differentiation medium in 15 ml tubes at 280 g for 5 min. The cells were incubated for 8 days for adipogenesis and 21 days for osteogenesis and chondrogenesis. Differentiation was subsequently evaluated by stainings with Oil Red O for adipogenesis, von Kossa for osteogenesis, and Masson's Trichrome for chondrogenesis. Differentiation was documented by light microscopy and the image areas positive for the respective stainings were quantified using ImageJ software for the adipogenic and chondrogenic differentiation, and AxioVision SE64 software for the osteogenic differentiation. The stained area was expressed as percentage of the total field of view for adipogenic and osteogenic differentiation. For chondrogenic differentiation, the area stained green was expressed as percentage of the total Trichrome-stained pellet.

#### 2.4.4 Cell migration assay

Cell migration was evaluated by seeding 50,000 cells/well in 6-well plates. At subconfluency, a scratch was made with a 1 mm pipette tip to create a cell-free area. Phase contrast images were taken immediately after the scratch was made, as well as 48 h later. Scratch closure was measured with ImageJ and quantified using the formula:

100 $$\text{Scratch}\;\text{closure}\;\%=\left(T1\;–\;T2\right)\;/\;T1\;*100$$

T1: Area immediately after scratch; T2: Area 48 h after scratch.

#### 2.4.5 Senescence assay

Senescence was measured using a Beta-galactosidase assay (catalog#: ENZ-KIT129-0120, Enzo Life Sciences (ELS) AG, Lausen, Switzerland) according to the manufacturer's instructions. MSCs were seeded at 1000 cells/well in a 96-well plate and incubated for 4 and 7 days. For analysis, cells were lysed on ice with a lysis buffer containing 0.5 % PMSF, centrifuged at 10,000×*g* for 10 min at 4 °C, and the supernatant was frozen at −80 °C. SA-β-gal activity was measured using a fluorescent substrate in a spectrophotometer at 360 nm (Excitation)/465 nm (Emission). Measurements for the mixed-fitness pool could not be performed due to limited reagent availability at the time of this experiment.

### 2.5 Tracking of cells in pooled cultures

#### 2.5.1 Cell labeling and fluorescence microscopy

The cells were tracked over time to analyze pool composition using fluorescence microscopy. Before pooling at passage 2 or 3, the cells from each donor in the pool were labeled with a specific fluorescent dye: Violet (CellTrace Violet, ThermoFisher Scientific GmbH), Far Red (CellTrace Far Red, ThermoFisher Scientific GmbH), and Green (Cytopainter, Abcam, Cambridge, UK). Next, an equal number of the labeled cells from each donor was combined (1:1:1) and the cells were seeded as the low, middle, high and mixed fitness pools. Fluorescence images were captured on days 1, 3, and 7 using a Nikon Ti2-E microscope.

For image quantification, we used an AI-based image analysis software (NIS.ai, Nikon), trained to detect and distinguish cells in the three fluorescence channels. The area covered by cells stained with each dye was measured, and the total cell-covered area was calculated. The contribution of each dye, i.e. donor, was expressed as percentage of the total.

#### 2.5.2 Genomic DNA markers and polymerase chain reaction

In addition to tracking the fluorochrome-labeled cells, the set-up of the mixed pool was designed to enable label-free cell tracking by genomic DNA (gDNA) markers. For this purpose, cells from all donors included in the study had been characterized for the presence or absence of specific gDNA markers. The mixed pool, besides considering the fitness scores, was then composed of MSCs from donors who were distinguishable based on their combination of the gDNA markers hGSTT1 and hSRY. For the cell tracking experiment, non-labeled cells from the mixed pool were harvested on day 7 and total gDNA was extracted from all samples using the DNeasy Blood & Tissue Kit (Qiagen, Hilden, Germany) with an additional RNase digestion step to eliminate RNA contamination, following the manufacturer's protocol.

Polymerase chain reaction was performed on 1 μg of gDNA using gene-specific primers from a published study [33] (Table 2), using iQ SYBR Green Supermix and a CFX Duet Real-Time PCR (Bio-Rad Laboratories Ges.m.b.H., Vienna, Austria). Fold change (FC) values were calculated using the Pfaffl formula. The respective gene-positive donor was set to 100 % as a baseline reference to represent the maximum contribution of that gene in the pool, based on which the proportions of each donor's DNA within the pooled sample were calculated. The results were normalized using the RHD gene, which was present in all donors, and the TUBA1A, which served as an internal control.

**Table 2:** Primer sequences.

	Forward primer	Reverse primer
SRY	GTTACCCGATTGTCCTACAGC	CGAAAGCCACACACTCAAGA
GSTT1	CCTCAGTGTGCATCATTCTCA	AAGTCCCAGAGCACCTCA
RHD	TCAAAGAGTGGCAGAGAAAGG	AACTTCCTCTCACTGTTGCC
TUBA1A	GGGCCAAGGCGACATCATTA	AGGTCATATCCCCAGCACGA

### 2.6 Statistical analysis

First, the data obtained from technical experimental replicates were averaged and mean values were used for further statistical analyses. Biological replicates, i.e. the individual MSC cultures, were used for statistical comparisons between fitness groups in the functional assays. The data from the respective pools were not included in this statistical analysis. For normally distributed datasets, analysis was done by ONE-way ANOVA with Bonferroni-adjusted post hoc tests using IBM SPSS Statistics 29 software (IBM Deutschland GmbH, Ehningen, Germany). If data were not normally distributed according to the Kolmogorov-Smirnov test, nonparametric tests were computed. Differences were considered significant at p < 0.05. Graphs were created in GraphPad Prism 10 and IBM SPSS Statistics 29 software. Scatterplots were used to visualize the data, with circles representing individual donor values including the median, while rhomb symbols were added manually in GraphPad Prism to indicate the pool data.

## 3 Results

### 3.1 Functional characterization of cells in individual and pooled cultures

#### 3.1.1 PDT and metabolic activity

All cell samples in the high-fitness group showed a rapid proliferation, with a short PDT and low variance within the data. Accordingly, metabolic activity, as measured by MTS assay, was overall highest in the high-fitness group and lowest in the low-fitness group. These differences became significant by day 7 (middle vs. low fitness: p = 0.016; high vs. low fitness: p = 0.014).

**Fig. 2:**
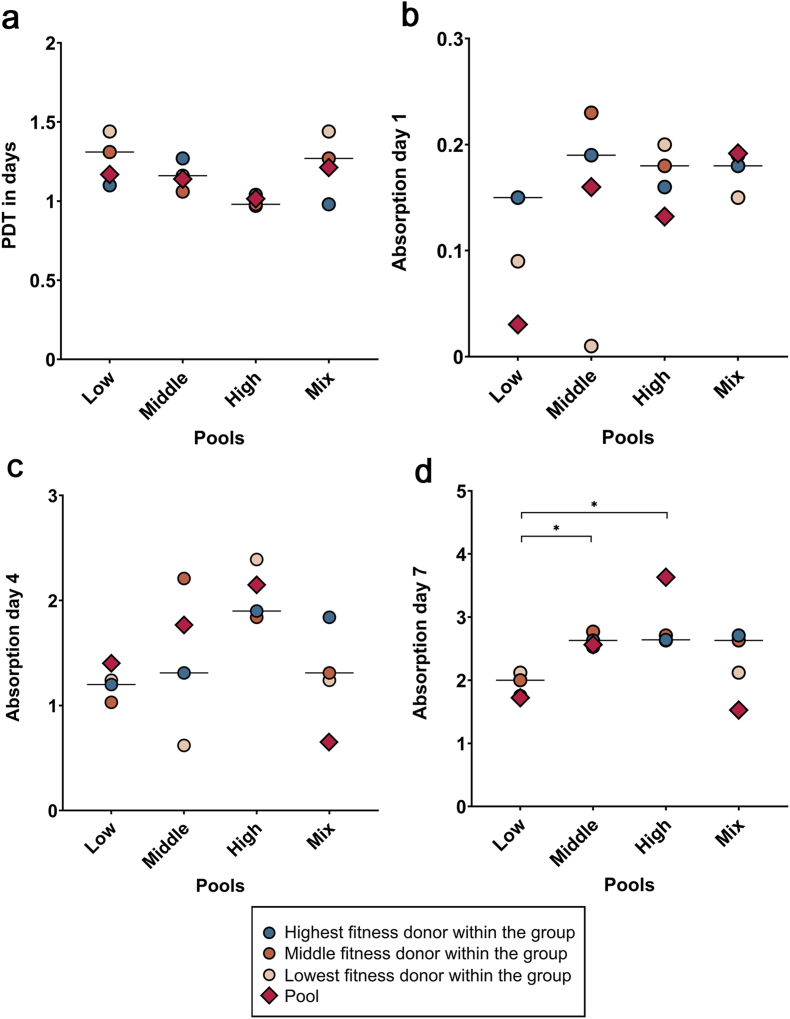
PDT calculated on day 5 for both individual and pooled settings (a), and metabolic activity on days 1 (b), 4 (c), and 7 (d). Data from the pools are displayed with the rhomb symbols in the scatterplots but were not included in the statistical analysis. Asterisks mark differences between the indicated groups with p < 0.05.

The PDT from the pooled cells aligned closely with the median values obtained from the individual donor cells in all fitness groups. However, the metabolic activity of the pooled cell cultures strongly differed from the median metabolic activity of individual cultures in both directions. For example, on day 7, the high fitness pool outperformed all high-fitness individual donors, while this was the opposite in the mixed fitness pool (Fig. 2).

#### 3.1.2 Trilineage differentiation

Adipogenic differentiation was indicated by the formation and accumulation of lipid droplets, confirmed by Oil Red O staining. Osteogenic differentiation was confirmed by the deposition of mineralization, with positive phosphate staining using von Kossa. Chondrogenic differentiation was verified through Masson's Trichrome staining, which demonstrated the deposition of collagens. Overall, multilineage differentiation potential was confirmed for all cell populations, but chondrogenesis in the mixed pool was very limited.

**Fig. 3:**
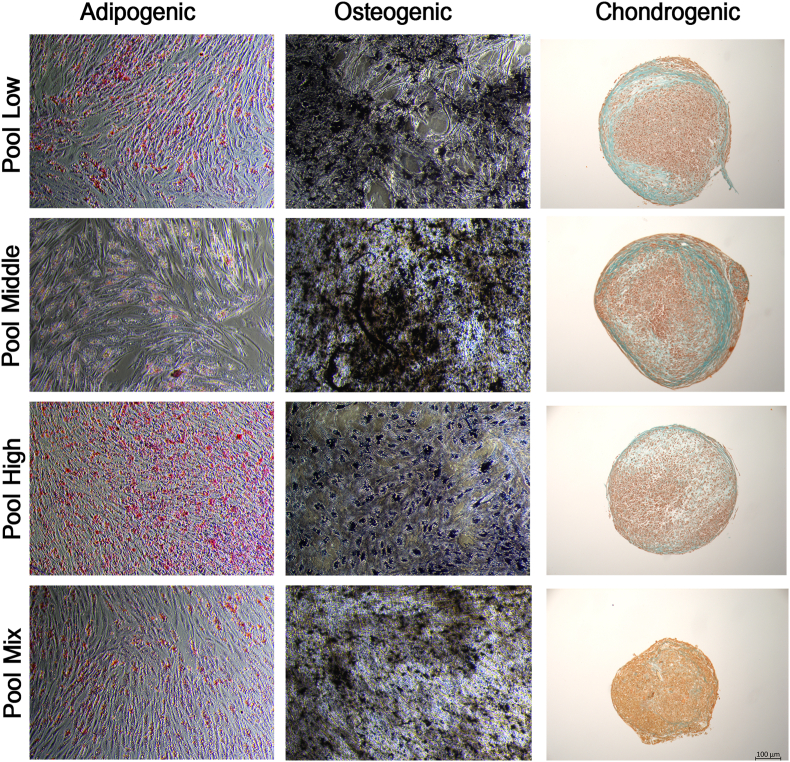
Trilineage differentiation. Pools shown as representatives for all the groups. Scale bar: 100 μm.

**Fig. 4:**
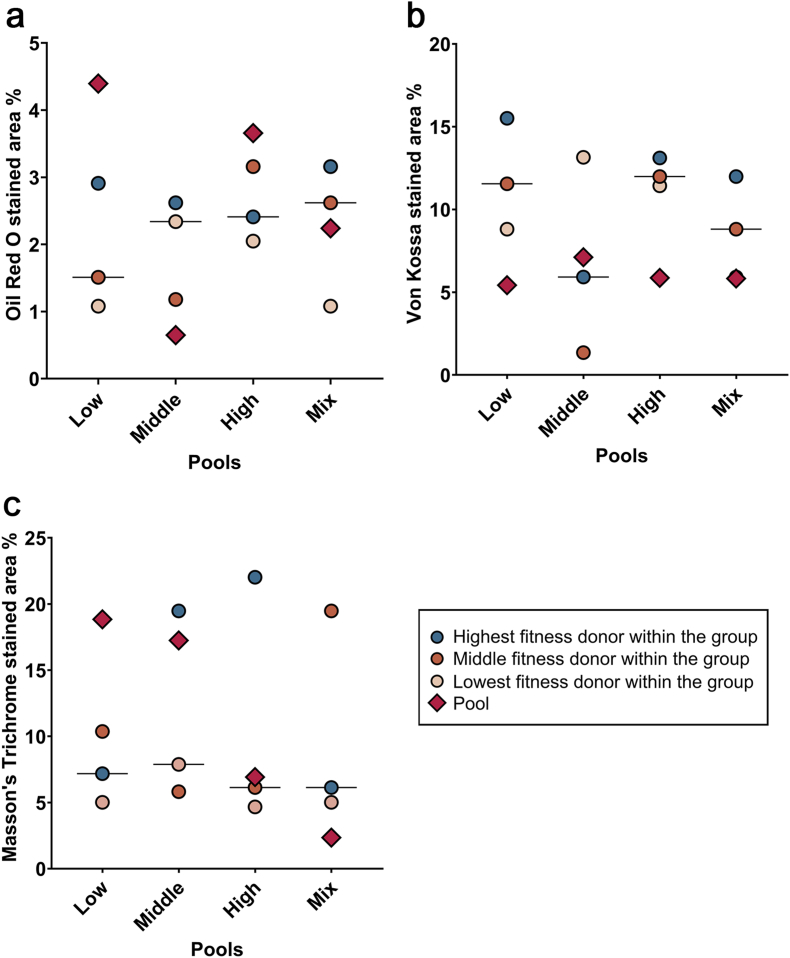
Trilineage differentiation. Area percentages positive for Oil Red O staining (a), von Kossa staining (b), and green staining within Masson's trichrome (c), as quantified with ImageJ following adipogenic, osteogenic and chondrogenic differentiation.

While the quantitative analysis showed no statistical significance between groups, differences were again noted between the individual donors’ results and pool results. Overall, the results obtained with the pooled cells often failed to be within the range of the results of the individual donor cells, with no consistent trend to perform superior or inferior (Fig. 3, Fig. 4).

#### 3.1.3 Migration potential

**Fig. 5:**
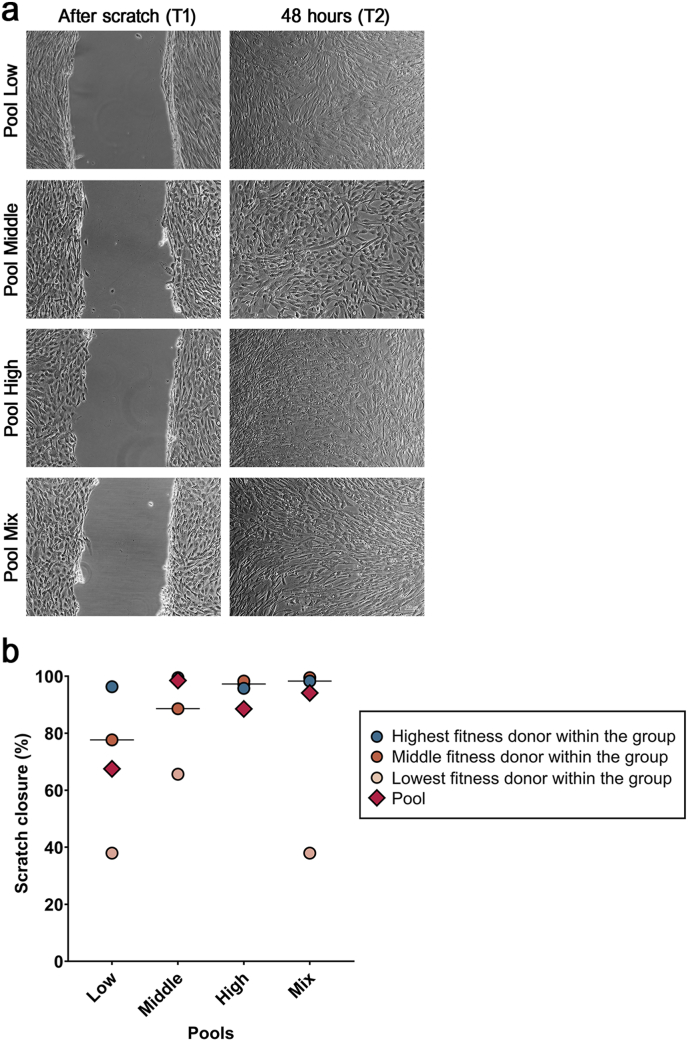
Scratch assay, with pools shown as representatives for their groups, right after the scratch was made (T1), and 48 h (T2) later (a) (scale bar: 100 μm), and scratch closure as quantified with ImageJ (b).

Migration capability was confirmed for all cell populations, with the scratch wounds being almost completely closed after 48 h. Differences between groups were not significant, and variability between individual donors was high in the low and mix-fitness groups but not in the high-fitness group. Again, the results obtained from the pooled cells did not always align well with the data from the individual donors (Fig. 5).

#### 3.1.4 Senescence

**Fig. 6:**
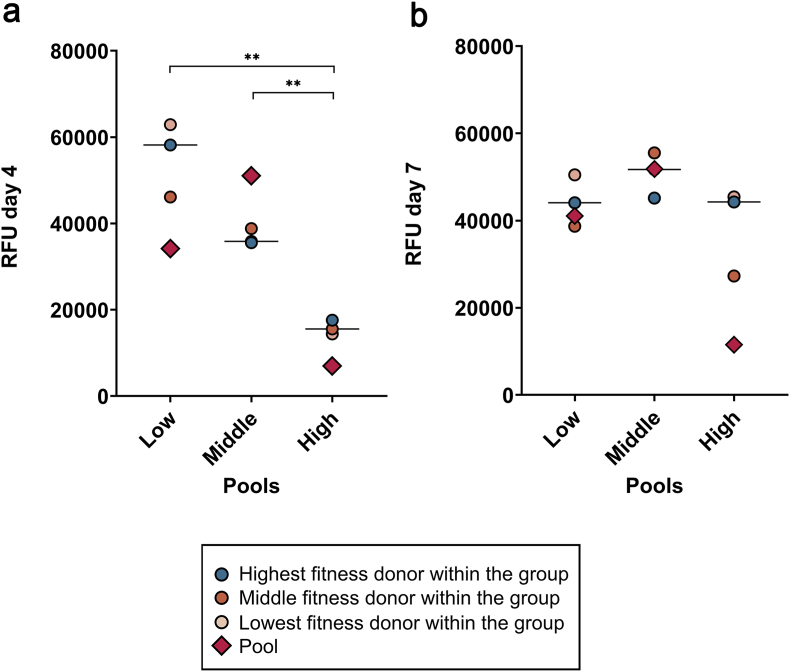
Senescence on day 4 (a) and day 7 (b), given in Relative Fluorescence Units (RFU). Data from the pools are displayed with the rhomb symbols in the scatterplots but were not included in the statistical analysis. Asterisks mark differences between the indicated groups with p < 0.01.

The senescence rates in the high-fitness group were lower compared to both the low-fitness (p = 0.01) and the middle-fitness (p = 0.008) groups on day 4. On day 7, differences were not significant. On day 7 but not on day 4, the results obtained from the pooled cells were roughly in line with the individual donors’ data, except for the high-fitness group on day 7, where the pool showed a lower senescence rate (Fig. 6).

### 3.2 Cell pool composition over time

**Fig. 7:**
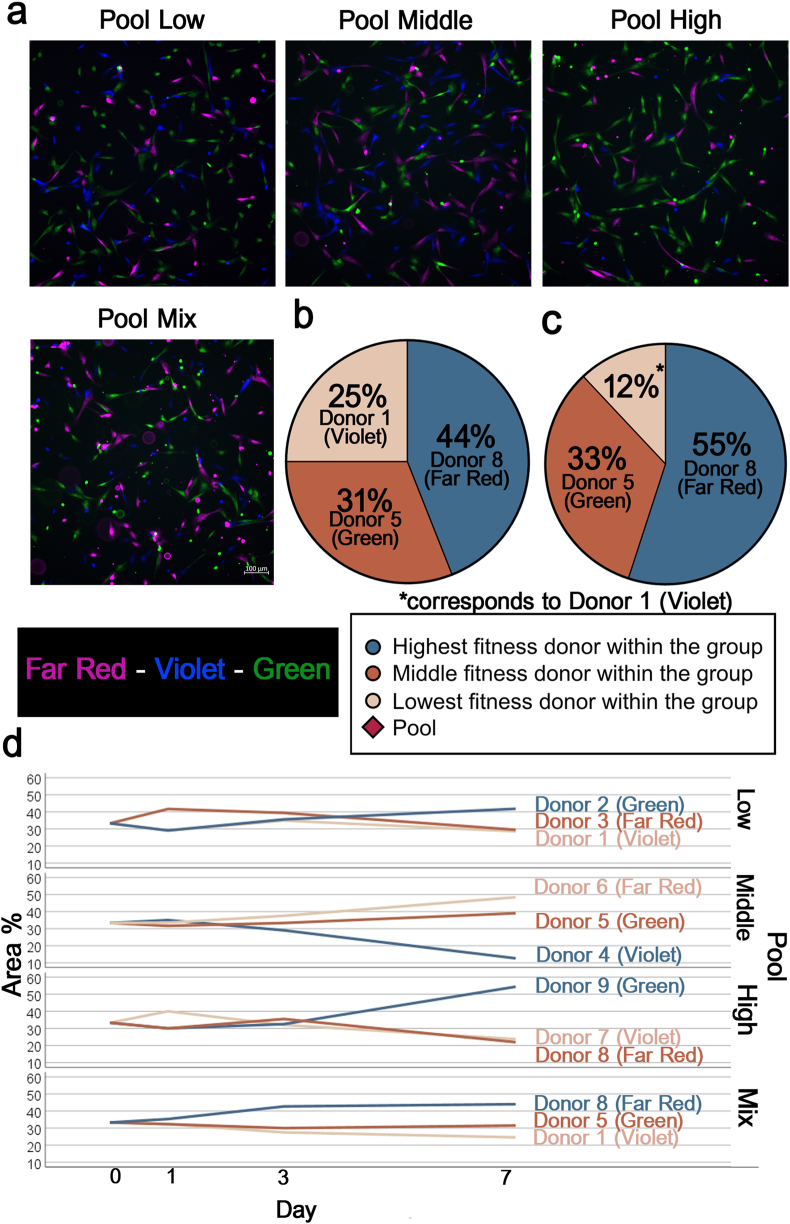
Pool composition over time. Representative images of the cell pools with individually labeled cells from each donor on day 1 (scale bar: 100 μm) (a). Donor contributions to the mixed-fitness pool after 7 days of culture, based on fluorescence microscopy analysis (b), and based on label-free tracking using qPCR analysis of gDNA markers (c). Changes in pool composition over time after composing the pools with equal proportions on day 0, as quantified by fluorescence microscopy analysis. Each line represents an individual donor, with donor numbers shown next to the lines and color-coded according to their assigned fitness group (d).

Quantification of fluorescence microscopy images demonstrated that the proportion of the cells from each individually labeled donor changed over time. By day 7, the mixed pool contained a higher percentage of the cells from the high-fitness donor (44 %) in comparison to the donors from the middle-fitness (31 %) and low-fitness (25 %) groups. These results were qualitatively confirmed by qPCR of donor-specific gDNA markers, although the exact percentages were not the same using this different approach (high-fitness donor: 55 %; middle-fitness donor: 33 %; low-fitness donor: 12 %) (Fig. 7).

Interestingly, however, such differences also occurred in the pools composed of similar fitness donors. All pools showed a dominant donor over time, with the donor with the highest cellular fitness in each respective group accounting for the largest percentage, except for the middle-fitness pool. In the high-fitness pool, the dominant donor contributed 54 %, and in the low-fitness pool, the dominant donor contributed 42 % (Fig. 7).

## 4 Discussion

In this study, we aimed to challenge the assumption that pooled MSC samples adequately average heterogeneous individual MSC properties. We investigated in how far individual MSCs, as defined by their cellular fitness, influence the functional behavior and composition of MSC pools. Our findings show that pooling MSCs does not offset donor variability; rather, pooled samples deviated notably from both the median and dominant donor, and different pools showed different results in the potency assays. We also showed that the composition of MSC pools already changes within one passage, influenced by even minor donor-specific cellular fitness differences, which disproportionately impacts the collective behavior of the pools. Our findings underscore the heterogeneity and complexity of MSC behavior, while highlighting that donor-specific characteristics and cellular fitness affect the composition and dynamics of MSC pools.

MSC heterogeneity, including donor-specific effects and the loss of potency during in vitro expansion, can impact therapeutic outcomes [21,34,35]. Therefore, it is crucial to understand how donor characteristics influence MSC biological behavior and clinical outcomes following MSC therapies [36]. For this reason, we decided to include low- as well as high-performing individual MSCs in the current study and to compile our cell pools from MSCs with distinct but defined fitness properties.

Prior to investigating MSC pooling, we determined the cellular fitness of individual donor MSCs by assessing their proliferation, colony formation and metabolic activity. Our results showed a relationship between cellular fitness and donor age, as expected and previously discussed in other studies [[37], [38], [39], [40]]. However, we also observed that cellular fitness does not solely correlate with age, underlining that other factors, such as donor health status, environmental conditions, or genetic factors, also play a significant role in determining cellular fitness [[41], [42], [43]]. As data protection regulations normally prevent researchers’ access to such information, and as their integration into a measure predictive of MSC fitness and potency represents an unsolved challenge, a thorough characterization of individual cell isolations remains crucial. However, so far, only one other study investigating pooled MSCs has pursued a similar approach, yet with a focus on immunological pre-characterization [44].

To explore the dynamics of MSC pools and their possible relationship with the fitness of the individual MSCs included, we created different cell pools based on the cellular fitness of the individual donors. When comparing the pooled MSCs to the individual donor MSCs in functional assays, we observed that pooled MSCs did not behave predictably. Although the population doublings of the pooled MSCs were in line with the respective individual donors, other functional data showed that pooling could lead to enhanced as well as compromised function, with no strict coherence between type of pool or type of assay. Still, it was noticeable that the mixed pool showed a lower metabolic activity and osteogenic and chondrogenic differentiation capacity than the relevant individual MSCs. These findings challenge the assumption that mitigating donor-specific differences by pooling is universally beneficial. The fact that data from our MSC pools did not align well with individual donor data is consistent with previous studies [28,30,31], in which a look into the data also reveals differences between individual MSCs and their respective pools. This could be used deliberately, as suggested by a recent study in which high-performing donors were included to increase overall pool immunomodulatory performance above the individual donors' average [44]. In that study, the pooled cells performed better than the individual donor MSCs’ average with respect to inhibition of T cell proliferation. This increased potency of the pool with respect to a specific function was achieved by pre-selecting the low-, medium- and high-performing donors included based on their performance in the same type of functional assay, rather than based on their overall fitness as done in the current study. However, other studies did not specifically address or explain existing differences between pools and individual cells [30,31], while still favoring the use of pooled MSCs as a basis for the product Stempeucel™ [45]. Considering the so-far unexplained differences observed between different pools and between pools and individual donors, while pooled MSCs offer advantages for clinical translation, it might be too early to draw simplified conclusions.

To our knowledge, no previous study has investigated pools with defined homogeneity versus defined heterogeneity, as addressed in the current study. Interestingly and in contrast to the hypothesis that the fittest donor within a pool will exert the main influence on pool functional properties, we observed that the overall performance in the mixed pool was lower than in the individual donors. This highlights that, while pooling may in some contexts enhance MSC function [25,44], it could also introduce drawbacks such as compromised differentiation potential. Furthermore, this observation aligns with the notion that MSC pools require time to adapt before acceptable results can be obtained, and our data suggest that this might be of particular relevance when cells with different fitness are combined. However, such adaptation after pooling requires prolonged cell expansion, which is known to negatively impact on MSC potency [46,47] and genetic stability [48,49], underlining that, at the very least, early pooling is crucial. Still, even if the doubling level can be kept low by early pooling, it is to be expected that the pool composition will change during the time allocated to adaptation.

To shed more light on the composition of MSC pools over the first week after pooling, we used two different complementary cell tracking techniques. Tracking cells without the use of gene editing to introduce artificial markers - which we considered important to avoid unpredictable alterations of the MSCs - comes with challenges. Therefore, we combined two different approaches to level out their respective advantages and disadvantages. Fluorescence microscopy has been widely used in studies to track cellular behavior [50,51]. It provided valuable insights into the donor-specific dynamics within the pools, as it allows for continuous monitoring of the cells over several days. However, fluorescent dyes can affect the viability of the cells, which may influence their behavior and present a challenge for long-term tracking. The robustness and toxicity of various vital stains has already been analyzed in other studies [52], where the issue of dye-related effects has been addressed to optimize the staining methods and to minimize these impacts. In our pilot experiments, we had specifically tested the tracer dyes used in the main experiments, and the results indicated that the fluorescent dyes, regardless of the specific stain, inherently affect cell viability to some degree. Importantly, no major differences in their impact on viability were observed between the dyes eventually used in our experiments. In contrast, tracking based on genomic markers avoids the viability issues associated with fluorescence dyes and offers a non-invasive alternative that preserves the physiological integrity of MSCs. Its own limitation was the difficulty with reliably distinguishing all donors due to overlapping genomic markers, making it impossible to analyze the composition of all four pools rather than only the mixed-fitness pool. These methodological differences, along with the challenges of both techniques, may explain the quantitative discrepancies in the results obtained from these two approaches. Nevertheless, the results from both techniques qualitatively entailed the same finding, namely the dominance of the fittest donor 7 days after pooling.

Our cell tracking data confirmed our hypothesis in that individual donor fitness significantly influences MSC pool composition, with the higher-fitness donors showing greater proliferation potential and thus, dominating the pool over time. Yet as described above, against our hypothesis, this dominance was rarely reflected in the functional assays and overall, pooling had variable effects on functional behavior. These dynamics highlight the complexity of MSC competition and reveal that the highest fitness donor does not necessarily drive overall favorable outcomes in pooled cultures. To better understand these dynamics, future studies should investigate factors that may influence MSC pool behavior, such as the communication between MSC from different donors via secreted factors and cell-cell contacts. Another aspect that could be explored in future studies is the stability of MSC surface markers (CD73, CD90, CD105) during pooled culture, as changes in their expression could indicate phenotypic drifts that might also affect functional behavior. Advanced single-cell analyses will need to disentangle the contributions of individual donors and provide deeper insights into the mechanisms driving these interactions.

With the current study, we aimed to address a critical gap in basic and preclinical MSC research by shedding light on how donor variability is overlooked in pooled cell models. This issue is fostered by simplified conclusions with incorrect phrasings. For example, previous reports concluded that pooling “reduces inter-donor variability” [44] or “reduces heterogeneity of different donors” [30]. This is technically not true, as inter-donor variability and heterogeneity of different donors is rather obscured than reduced, while only the variability of the obtained data is actually reduced. Importantly, in our approach, the data obtained with pooled MSCs neither proportionately reflected the properties of all included individual MSCs, nor the properties of the fittest included MSCs. However, so far, only few studies specifically addressing the effects of MSC pooling exist, thus several relevant aspects remain to be covered, such as different MSC sources or diverse types of functional assays.

## 5 Conclusion

In conclusion, our study underlines that further work is crucial to understand cell pool dynamics and that the impact of pooling on specific MSC functions requires careful further investigation to advance personalized therapies based on pooled cells. Moreover, the observed discrepancy between pooled and individual donor data strongly emphasizes the importance of biological replicates. They are essential for capturing the full range of donor variation, ensuring that cell culture models are representative of natural diversity.

## Author contributions

Concept and study design: JB, SN, DK; acquisition of tissue samples: CB; cell culture experiments: DK; acquisition and analysis of data: DK, SN, CS, UR; interpretation of data: DK, SN, JB; manuscript draft and revision: DK, JB, SN. All authors have critically revised the manuscript and approved the submitted version.

## Data availability

The data are presented within the manuscript and the supplementary material. Additional supplementary data are available from the authors on request.

## Funding

This project was funded by the Hessian Ministry of Science and Research, Arts and Culture, Hesse, Germany, as LOEWE Exploration research grant (III L5 - 519/06/00.002-(0012)).

## Declaration of competing interest

The authors declare that the research was conducted in the absence of any commercial or financial relationships that could be construed as a potential conflict of interest.
