# Supplementary material for: No simple way to averaging out: Pooled mesenchymal stromal cells do not reflect average donor characteristics
Source: Regen Ther. 2025 Oct 3;30:849–59. doi: 10.1016/j.reth.2025.09.012 (PMC12519293; doi:10.1016/j.reth.2025.09.012)
Supplement: Multimedia component 1 [file mmc1.pdf]

Supplementary Material to Kukaj et al.:

Fitness scoring in MSCs from individual donors

Parameter/ assay	Data	Scoring range	Comment
Population doubling	Population doubling time (PDT)	1-9	Lower PDT = higher fitness score
Metabolic activity (MTS)	Absorbance	1-9	Higher absorbance = higher fitness score
Colony-forming potential (CFU)	Plating efficiency (PE)	1-9	Higher PE = higher fitness score
Total fitness score	Composite score (sum)	3-27	Used for donor ranking and pool assignment

Table S1. Cellular fitness scoring approach.

Individual MSCs were subjected to population doubling, metabolic activity and colony forming unit assays as described in the main manuscript (2.3). Based on the results obtained, for each parameter, a score between 1 and 9 was assigned. The score points for all parameters were summed up to obtain the total fitness score for each individual MSC sample.

Supplementary Material to Kukaj et al.:

Fitness scoring in MSCs from individual donors

Donor	PDT (days) [score]	MTS (absorption) [score]	CFU (PE%) [score]	Total fitness score	Fitness group
**1**	2.18 [4]	1.33 [3]	3.29 [1]	8	Low*
**2**	2.17 [5]	1.03 [2]	3.99 [2]	9	Low
**3**	1.65 [7]	0.95 [1]	4.51 [3]	11	Low
**4**	2.46 [2]	2.73 [9]	7.81 [5]	16	Middle
**5**	2.42 [3]	1.81 [6]	9.55 [7]	16	Middle*
**6**	2.82 [1]	1.65 [5]	9.55 [8]	14	Middle
**7**	2.15 [6]	2.10 [7]	5.90 [4]	17	High
**8**	1.64 [8]	1.46 [4]	8.68 [6]	18	High*
**9**	1.61 [9]	2.16 [8]	9.72 [9]	26	High

Table S2. Assay results and resulting fitness scores.

The table presents the cell fitness assay results for each donor, including population doubling time (PDT; given as the mean from the different passages), metabolic activity/ MTS assay absorption, and colony forming potential/ plating efficiency (PE), as well as the score points assigned for each assay and donor. Donors were grouped into low, middle, and high cellular fitness categories based on their total fitness scores.

Low, middle and high fitness pools were then composed by combining three donors per group, along with a mixed pool comprising one donor from each category. Donors marked with an asterisk (*) were also included in the mixed-fitness pool.
